# Diaphragm Atrophy and Contractile Dysfunction in a Murine Model of Pulmonary Hypertension

**DOI:** 10.1371/journal.pone.0062702

**Published:** 2013-04-22

**Authors:** Bumsoo Ahn, Hyacinth M. Empinado, Monsour Al-Rajhi, Andrew R. Judge, Leonardo F. Ferreira

**Affiliations:** 1 Department of Applied Physiology and Kinesiology University of Florida, Gainesville, Florida, United States of America; 2 Department of Physical Therapy, University of Florida, Gainesville, Florida, United States of America; University of Texas Health Science Center at Houston, United States of America

## Abstract

Pulmonary hypertension (PH) causes loss of body weight and inspiratory (diaphragm) muscle dysfunction. A model of PH induced by drug (monocrotaline, MCT) has been extensively used in mice to examine the etiology of PH. However, it is unclear if PH induced by MCT in mice reproduces the loss of body weight and diaphragm muscle dysfunction seen in patients. This is a pre-requisite for widespread use of mice to examine mechanisms of cachexia and diaphragm abnormalities in PH. Thus, we measured body and soleus muscle weight, food intake, and diaphragm contractile properties in mice after 6–8 weeks of saline (control) or MCT (600 mg/kg) injections. Body weight progressively decreased in PH mice, while food intake was similar in both groups. PH decreased (*P*<0.05) diaphragm maximal isometric specific force, maximal shortening velocity, and peak power. Protein carbonyls in whole-diaphragm lysates and the abundance of select myofibrillar proteins were unchanged by PH. Our findings show diaphragm isometric and isotonic contractile abnormalities in a murine model of PH induced by MCT. Overall, the murine model of PH elicited by MCT mimics loss of body weight and diaphragm muscle weakness reported in PH patients.

## Introduction

Pulmonary hypertension (PH) is a chronic, progressive disease characterized by inflammation and pulmonary vascular remodeling leading to high blood pressure in the pulmonary circulation [1]. Symptoms of PH include breathlessness, weakness, and fatigue. These symptoms have been traditionally attributed to pulmonary gas exchange abnormalities [2]. However, important factors in PH symptomatology that have emerged in recent years are cachexia and skeletal muscle weakness [3], [4]. Inspiratory muscle weakness has been documented in PH patients [5]. The diaphragm is the primary muscle used during inspiratory breathing efforts; hence, depression of diaphragm force is of particular relevance for symptoms of breathlessness and fatigue experienced by patients with PH. Depression of muscle force can result from atrophy (decrease in fiber size or cross-sectional area) and contractile dysfunction (defined as force normalized for muscle cross-sectional area). Patients with PH also experience loss of body weight [4], which is associated with poor prognosis in chronic diseases, e.g., heart failure [6]. However, diaphragm weakness and loss of body weight remain poorly understood in PH.

Diaphragm weakness in PH results from loss of muscle fiber size (atrophy) and contractile dysfunction [3], [7]. Contractile dysfunction has been shown by lower maximal diaphragm isometric tension normalized for cross sectional area in rats and humans with PH [7]–[9], whereas the effects of PH on diaphragm isometric force in a mouse model of PH remains unknown. In addition, impaired ventilatory function and diminished inspiratory pressure during dynamic maneuvers suggest diaphragm isotonic contractile impairments in patients with PH, but could also be due to abnormalities extrinsic to the diaphragm muscle (e.g., neurological or airway narrowing). However, it is unclear whether PH impairs diaphragm isotonic contractile properties.

Monocrotaline (MCT) has been widely used to induce PH and right ventricular (RV) failure in rodents [10]–[15]. To our knowledge, studies focusing on skeletal muscle abnormalities caused by PH and RV failure induced via MCT have been done solely in rats [8], [14], [16], [17]. Rats are hypersensitive to MCT – 10 times more sensitive than mice [12] – and become severely anorexic upon exposure to MCT [14]. While PH causes loss of body weight [4], it appears that PH per se does not necessarily cause anorexia in humans [18], [19]. Mice receiving MCT develop PH and show a progressive loss of body weight like rats [15], [20], but it is unknown if mice receiving MCT are anorexic. This aspect is relevant for further understanding diaphragm abnormalities in rodent models of PH.

The primary purpose of the present study was to investigate the effects of PH on diaphragm fiber atrophy and contractile properties in the mouse model of the disease induced by MCT. We found that PH caused diaphragm fiber atrophy and impaired isometric and isotonic contractile function in mice. As potential mechanisms for contractile dysfunction in chronic diseases, we tested for protein oxidation and selective degradation of myofibrillar proteins and found those variables were unaffected by the disease. A secondary purpose of our study was to examine changes in body weight and food intake in mice treated with MCT. We observed that progressive loss of body weight in PH mice occurred in the absence of anorexia.

## Methods

### Animals and procedures

We strictly followed all recommendations for the care and use of laboratory animals established by the National Institute of Health [21]. The study was also approved by the Institutional Animal Care and Use Committee at the University of Florida (protocol # 201105762), and all efforts were made to minimize animal suffering. We used C57BL6 male mice aged 8–12 wks old at the beginning of the experiments (Harlan, Indianapolis, IN).

Mice were individually caged, exposed to a 12∶12 hr dark:light cycle, and had access to standard water and chow *ad libitum*. We administered weekly injections of 600 mg/kg MCT (20 μL/g body weight, subcutaneous) or equivalent volume of sterile saline for 6–8 weeks to induce PH as shown by Yamazato et al. [15]. We measured body weight and food consumption 2–3× per week. On the day of the experiment, we anesthetized the mice with subcutaneous injection of ketamine (100 mg/kg) and xylazine (15 mg/kg). When the animals reached the surgical plane of anesthesia, we performed a laparotomy to excise diaphragm, heart, and lungs. One hemidiaphragm was quickly frozen in liquid nitrogen for biochemical analyses. The other hemidiaphragm was used to for histology and to measure contractile properties *in vitro* (see below). The heart was dissected to remove atria, major vessels, and separate the right ventricle (RV) from left ventricle plus septum (LV+S). Lungs, RV, and LV+S were blotted dry and weighed. We also removed soleus and blotted dry prior to measuring weight.

### Histology

We used standard procedures for tissue preparation as outlined elsewhere [22], [23]. Briefly, diaphragm bundles were were embedded in Tissue-Tek freezing medium, frozen in liquid-nitrogen-cooled isopentane, and stored at −80°C. We sliced diaphragm bundles into 10 μm cross-sections using a Microm HM 550 cryostat (Microm International, Walldorf, Germany). Sections were incubated in 0.5% Triton X-100 solution for 5 minutes at room temperature, then washed in PBS for 5 minutes. Sections were subsequently incubated in anti-Laminin primary antibody (1∶200; Sigma-Aldrich) for 1 hour at room temperature in a humid chamber. After primary antibody incubation, sections were washed in PBS 3× for 5 minutes and incubated in anti-Rabbit rhodamine red secondary antibody (1∶100; Sigma-Aldrich) in blocking solution for 1 hour at room temperature in a humid chamber. Sections were then washed 3× for 5 min in PBS. We used a Zeiss Axio Observer-A1 microscope (Carl Zeiss Microscopy, Jena, Germany) and AxioCam MRm3 camera to capture images and NIH ImageJ software for image analysis. We traced the cell membrane envelope delineated by laminin to measure the area of all visible fibers from each diaphragm muscle section.

### Isolated diaphragm contractile properties

We dissected a diaphragm strip along with rib and central tendon in bicarbonate-buffered solution (in mM: 137 NaCl, 5 KCl, 1 MgSO_4_, 1 NaH_2_PO_4_, 24 NaHCO_3_, 2 CaCl_2_) gassed with a mixture of 95% O_2_ and 5% CO_2_ at room temperature. We used 4.0 silk suture to tie the rib to a glass rod and to attach the central tendon to a Dual-Mode Muscle Lever System (300C-LR, Aurora Scientific Inc., Aurora, Canada). The diaphragm strip was placed in an organ bath containing bicarbonate-buffered solution. We adjusted bundle length to attain maximal twitch tension (optimal length, Lo), increased the temperature of the organ bath to 37°C, added D-tubocurarine (25 μM) to the solution, and after thermo-equilibration (20 min) started our force-frequency protocol. The isometric force-frequency protocol consisted of pulse frequencies of 1–300 Hz interspersed by 1 min intervals. The stimulation protocol consisted of supramaximal electrical current (600 mA) delivered through platinum electrodes using a biphasic high-power stimulator (701C, Aurora Scientific Inc.) with pulse duration of 0.25 ms and 300 ms train duration.

To determine the force-velocity relationship, we used afterloaded contractions employing a protocol similar to previous studies [24]–[26]. In this protocol, the bundle was maximally stimulated (300 Hz) to shorten against an external load corresponding to 2–80% P_o_ above the resting tension. Each stimulus lasted for 200 ms and was delivered with 2 min intervals. We measured velocity at least 10 ms after the initial change in length in the linear portion of the tracing. We normalized shortening velocity per optimal length (L_o_) and force per cross-sectional area (CSA, kN/m^2^). To estimate the bundle CSA, we divided diaphragm bundle weight (g) by bundle length (cm) multiplied by muscle specific density (1.056) [27]. The force-velocity curve was plotted and fitted to the Hill equation [28]. We determined maximal shortening velocity (V_max_) as the velocity at zero force in the force-velocity relationship. Power output for each isotonic contraction was also calculated as Force × Velocity and is given in kN/m^2^ × Lo/s.

### Preparation of diaphragm tissue lysates

We homogenized diaphragm samples on ice using Kontes Duall Tissue Grinders in muscle extraction buffer (20 mM Hepes; pH 7.4, 2 mM EGTA, 1% Triton-X100, 50% Glycerol, 50 mM β-Glycerophosphate, 1× Protease Inhibitor Cocktail, 1× Phosphatase Inhibitor Cocktail (Thermoscientific)) [29]. Homogenates were rotated end over end for 1 hr at 4°C and then sonicated. The samples were then centrifuged at 15,000 *g* for 2 min at room temperature to pellet insoluble debris. We kept the supernatant and determined its protein content using the DC protein assay (Bio-Rad Laboratories).

### Immunoblotting

We loaded 10–15 μg of protein into 4–15% polyacrylamide gradient gels (Bio-Rad Laboratories) and performed electrophoresis at 200 V for 50 mins at room temperature, and then transferred proteins to low fluorescence PVDF membranes (Immobilon-FL) at 100 mA overnight at 4°C. We blocked the membranes in either Odyssey Blocking Buffer (LI-COR) for 1 hr at room temperature, and probed with a sarcomeric actin specific antibody (JLA 20; Developmental Studies Hybridoma Bank, University of Iowa, Iowa City, IA) and a anti-troponin T1-T2-T3 antibody (TT-98; AbCam) diluted 1∶1000 and incubated overnight at 4°C, followed by secondary antibodies (IRDye, LI-COR) diluted 1∶10,000 and incubated for 45 minutes at room temperature. We quantified the bands using Odyssey Infrared Imaging system (LI-COR, Lincoln, NE). We stripped the membranes using 1× stripping buffer (LI-COR) for 20 minutes, re-probed with α-tubulin antibody (Developmental Studies Hybridoma Bank), and determined the abundance of α-tubulin, which was used for normalization purposes.

### Protein Carbonyls Assay

We measured protein carbonyls in whole diaphragm homogenates using the Oxyblot kit according to manufacturer's instructions (Millipore Corporation). Briefly, we reacted 20 μg of protein with 2,4 dinitrophenylhydrazine (DNPH) and immunoblotted (see above) using anti-DNP primary antibody and anti-rabbit secondary antibody (IRDye 800CW). We scanned the membranes and quantified integrated intensity in each lane using the Odyssey Infrared Imaging system (LI-COR). We stripped the membranes using 1× stripping buffer (LI-COR) for 20 minutes, re-probed with α-tubulin antibody (Developmental Studies Hybridoma Bank), and determined the abundance of α-tubulin. Protein carbonyls were normalized for α-tubulin.

### Myofibrillar protein isolation [30]

We homogenized diaphragm samples with Kontes Duall Tissue Grinders in Standard Relax Buffer with Triton X-100 (SRB-X100; 75 mM KCl, 10 mM imidazole, 2 mM MgCl_2_, 2 mM EDTA, 1 mM NaN_3_, 1% v/v Triton X-100), and then centrifuged for 1 minute at 20,000× g at 4°C. We washed the pellet once with SRB-X100 and once with SRB (75 mM KCl, 10 mM imidazole, 2 mM MgCl_2_, 2 mM EDTA, 1 mM NaN_3_) centrifuging at 20,000× g for 1 min at 4°C and discarding the supernatant each time. The pellet was then resuspended in 1∶20 (w/v) SDS-Page industrial buffer (8M urea, 2M thiourea, 0.05M Tris pH 6.8, 75 mM DTT, 3% SDS), vortexed for 1 h and centrifuged at 20,000 g for 10 min at room temperature. The supernatant containing the clarified soluble sample is the myofibrillar protein enriched fraction and was stored at −80°C. We determined protein concentrations of the myofibrillar protein enriched fraction using the RC DC Assay (Bio-Rad, Hercules, CA). We loaded 15 μg of protein into a 4–15% polyacrylamide gradient gel (Criterion precast gels; Bio-Rad, Hercules, CA), protein molecular weight standards (Precision Plus, Bio-Rad), and subjected to gel electrophoresis for 55 min at 200 V at room temperature. The gel was stained with Coomassie blue overnight (Thermo Fisher Scientific, Waltham, MA) and washed with dH_2_O three times. We quantified the optical density of individual bands and the whole lane (total protein) using the Odyssey Infrared Imaging system (LI-COR, Lincoln, NE) to determine the abundance of specific and total myofibrillar proteins. Specific proteins of interest were identified based on their known molecular weights, the band was quantified and data were normalized to total protein.

### Statistical analysis

Data are shown as mean ± SE. Statistical analyses were conducted using either Student's t-tests or two-way repeated-measures ANOVA followed by Bonferroni *post hoc* test (Prism 5.0b, GraphPad Software Inc., La Jolla, CA). We declared statistical significance when *P*<0.05.

## Results

We administered MCT to a total of 14 mice. Of those, 2 animals died spontaneously and 2 were euthanized due to poor health conditions as recommended by institutional veterinarians. Thus, data from a total of 10 mice that survived the entire duration of the study (6–8 weeks) are reported here. In the cases of spontaneous death or euthanasia, the profile of changes in body weight resembled that of animals surviving the 6–8 weeks of treatment.

Several laboratories have used MCT to induce PH in mice [12], [13], [20]. The protocol we used has been shown to promote a 2.5 fold increase in right ventricular systolic pressure (RVSP) in C57BL6 mice over 8 weeks [13], [15], [20]. In this setting, PH causes RV hypertrophy defined by an increase in the ratio of RV weight to LV+S weight [RV/(LV+S)] of ∼20–25%. Due to technical problems, we were not able to measure RVSP, but MCT increased RV/(LV+S) by ∼18±2% in our study. We also observed other typical signs of PH after 6–8 weeks of MCT injections, such as increased RV and lung weight (Table 1). Hence, we consider that mice in the MCT group developed PH consistent with previous studies [13], [15], [20] and, heretofore, refer to this group as PH for clarity.

**Table 1:** Body and tissue weight in control and pulmonary hypertension mice.

	Control	PH
Body weight (g), pre-treatment	28±0.7	28±0.4
Body weight (g), post-treatment	29±0.9	25±0.7 *
Soleus weight (mg)	10.0±0.3	8.5±0.3 *
Right ventricle weight (mg)	22.6±0.8	26.1±0.9 *
Left ventricle weight (mg)	99.6±2.6	98.4±2.6
RV/(RV+LV)	0.18±0.01	0.21±0.01 *
Lung weight (mg)	135±4.3	163±6.5 *

Abbreviations: PH, pulmonary hypertension; RV, right ventricle; LV, left ventricle weight. Data are mean ± SE from mice receiving saline (control) or monocrotaline injection for 6–8 weeks. Mice receiving monocrotaline have typical signs of PH such as increased RV and lung weight, and RV/(RV+LV) weight. N = 10–16 (Control) and 5–9 (MCT). * *P*<0.05 different from the control group.

**Figure 1:**
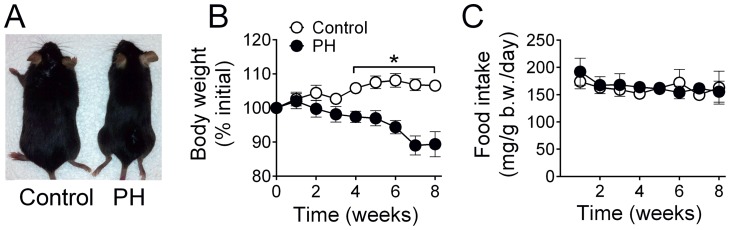
Progressive decrease in body weight and unchanged food intake in mice treated with monocrotaline to induce pulmonary hypertension (PH). A) Images of sample mice in each group at the end of study illustrating that PH mice are smaller than controls. B) Body weight expressed as percentage of value at the onset of study. C) Food intake expressed as mg per g body weight per day in each week of the study period. Data are from mice receiving control and monocrotaline injections for 8 weeks (N = 4 for Control; N = 5 for PH). * *P*<0.05 for control vs. PH.

PH caused a progressive decrease in body weight after the first week of treatment (Figure 1). The decrease in body weight was accompanied by ∼15±1% lower soleus weight (Table 1). Importantly, food intake normalized for body weight was similar between groups and unchanged over the course of 8 weeks (Fig. 1C). The outcome was the same when absolute food intake was analyzed as average food intake during the 8-week period was 4.2±0.2 g/day for control and 4.3±0.4 g/day for PH.

**Figure 2:**
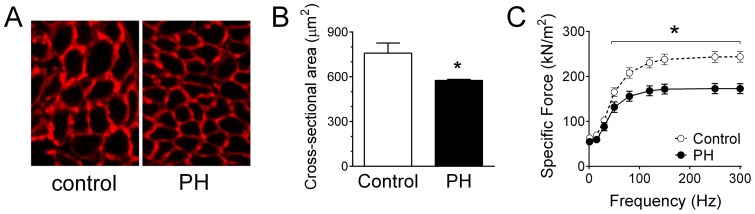
Diaphragm fiber cross sectional area and isometric force are decreased in mice with pulmonary hypertension (PH). A) Cross sections taken from diaphragm muscle fiber bundles of sample control and PH mice. B) Diaphragm fiber cross sectional area of 200–300 fibers per muscle, from 3–4 mice per group. C) Specific Force, force normalized to bundle cross-sectional area. Data are from diaphragm bundles of controls and mice receiving monocrotaline for 6–8 weeks to induce PH (N = 10/group). * P<0.05 for PH vs. control.

We conducted morphological analysis in cross-sections of diaphragm bundles and measured diaphragm function in isolated bundles *in vitro*. Fiber cross-sectional area was decreased by 25% in PH mice (Fig. 2A–B). Due to problems in trials of RVSP measurements (see above), we were unable to determine diaphragm contractile properties in 4 saline-injected mice. Saline injections had no effect on diaphragm contractile properties when compared to data from age-matched mice. Thus, we included in our control group data of diaphragm function from age-matched mice that did not receive injections. PH decreased submaximal and maximal specific isometric tetanic force by 25–30% (Fig. 2C). Twitch force was unchanged (6.3±0.4 N/cm^2^ for control; 5.5±0.45 for PH) and, as a consequence, the twitch-to-tetanus ratio increased in PH. Time to peak tension (14±0.5 ms for control; 15±1 ms for PH) and one half relaxation time (17±1.1 ms for control; 17±1.4 ms for PH) did not differ between groups.

**Figure 3:**
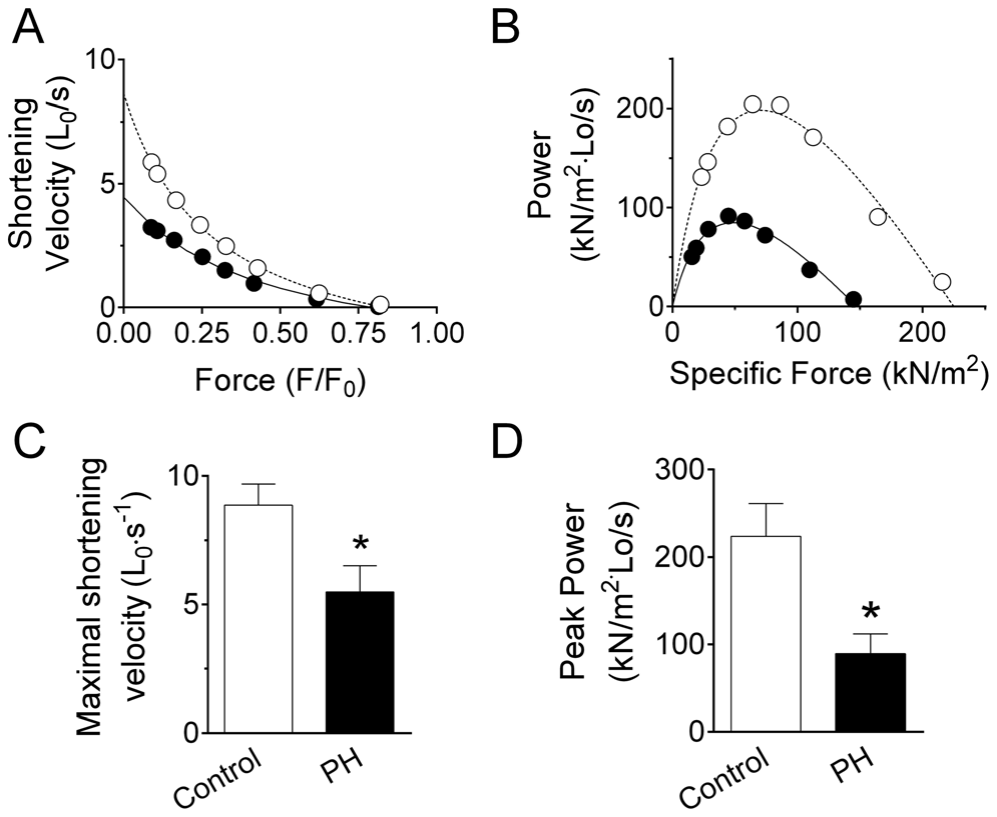
Pulmonary hypertension impairs isotonic contractile properties of isolated diaphragm bundles. A) Force-velocity relationship from sample control (open circles) and PH mice (closed circles). Force is relative to maximum isometric tetanic force (*F*_o_). Shortening velocity is normalized to optimal bundle length for twitch force (L_0_). Solid and dashed lines are best fit from Hill equation. B) Maximal shortening velocity (V_max_) determined from extrapolation of shortening velocity to zero force using Hill equation (see Panel A for example). C) Force-power relationship of sample control and PH mice. Power is calculated as specific force (kN/m^2^) multiplied to shortening velocity (L_0_/s) shown in panel A. D) Peak Power from all mice in each group. Data are mean ± SE. Data in panels C and D are from n = 6/group. * *P*<0.05 vs. control.

An important and novel observation was that PH impaired isotonic contractile properties in the diaphragm. Specifically, maximal shortening velocity (V_max_) was 40% slower and peak power was 63% lower in PH than control (*P*<0.05; Fig. 3), despite a 138% increase in curvature of the force velocity relationship. The differences in the force-velocity characteristics remained after normalizing for P_o_ (Fig. 3A).

**Figure 4:**
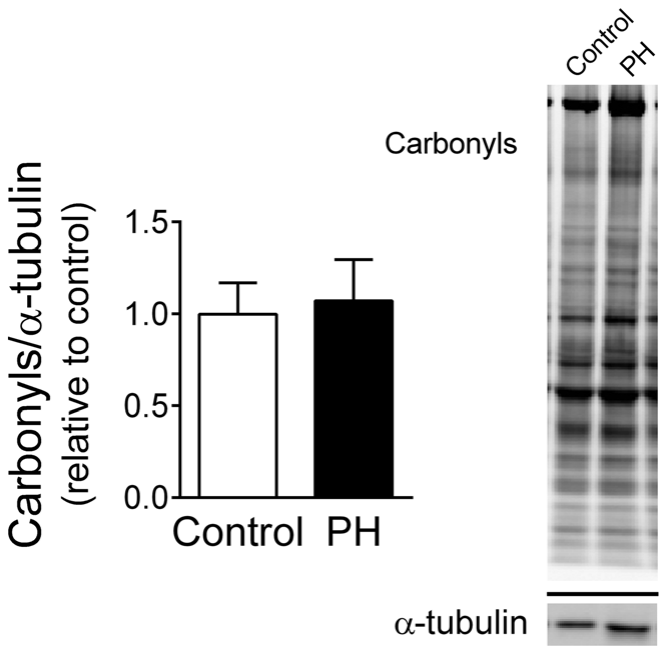
Protein carbonyls are unchanged in diaphragm from PH mice. These findings suggest that PH does not increase protein oxidation in mouse diaphragm. Total protein carbonyls are normalized to corresponding α-tubulin and expressed relative to the mean for the control group (n = 4/group). Image shows membranes probed for protein carbonyls and α-tubulin. Lanes are examples of control and PH diaphragm.

Based on our findings of impaired isometric and isotonic diaphragm contractile function, we examined post-translational modifications of myofibrillar proteins as potential mechanisms of dysfunction. We measured total protein carbonyls as marker of oxidation and found no change in the diaphragm of PH mice (Fig. 4). Nor were there changes in the abundance of carbonyls in individual bands (data not shown).

**Figure 5:**
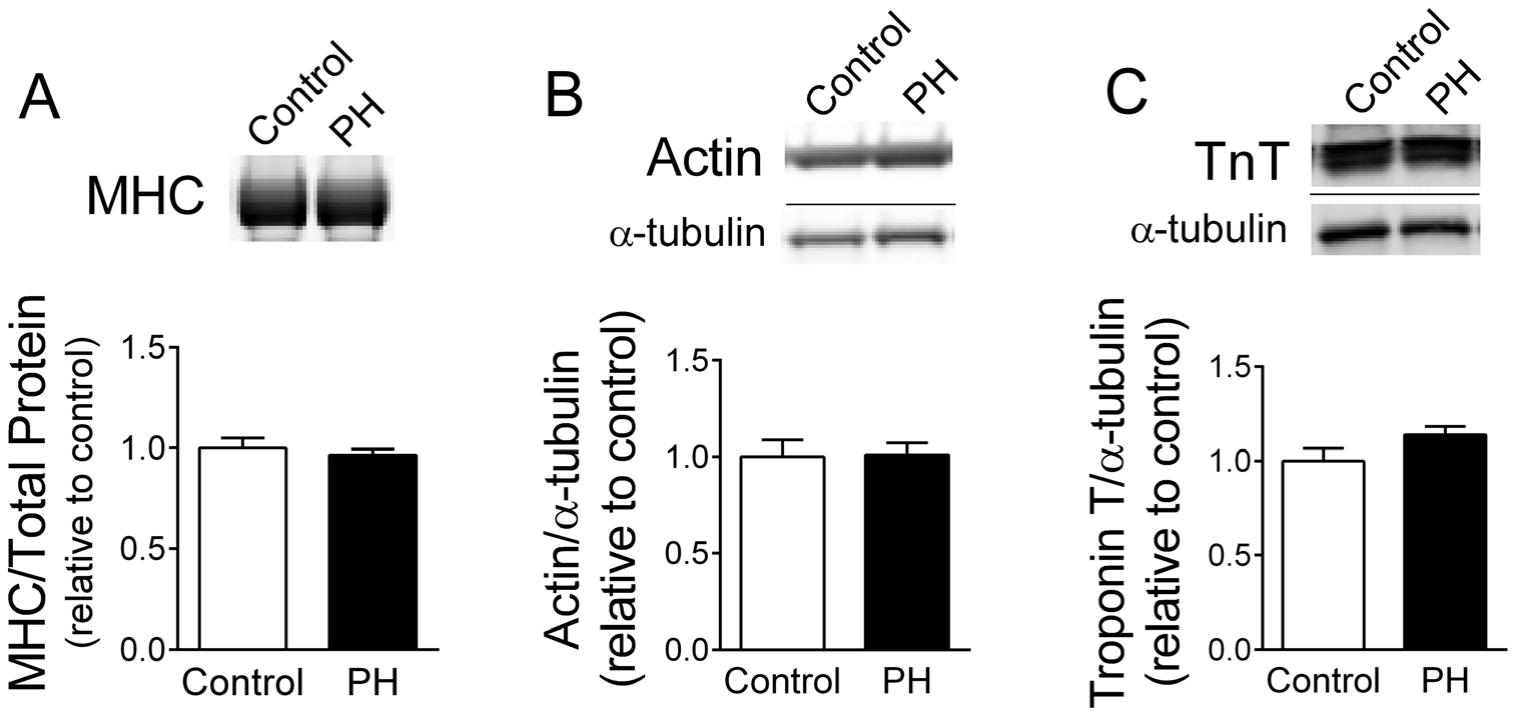
Myofibrillar protein abundance is similar in control and PH mice. *A*) Images show protein bands corresponding to myosin heavy chain (∼220 kDa) in examples of control and PH mice. MHC data were normalized for total protein measured as optical density of entire lane. Molecular weights were determined from protein standards (not shown). *B and C*) Images are examples of lanes from membranes probed for sarcomeric actin (*B*) and troponin T (*C*) in whole-muscle homogenates. Optical density of actin and troponin T were normalized to α-tubulin in the respective membranes. All bar graphs show mean ± SE of results expressed relative to mean of control (N = 4/group).

Protein content measured by the RC-DC assay in myofibrillar protein enriched fractions was similar in control (1.20±0.17 mg/ml and PH (1.10±0.38 mg/ml). Based on the existing literature, we focused our analysis of myofibrillar protein abundance on MHC, actin, and troponin T. We found that the optical density of the band corresponding to myosin heavy chain (MHC, ∼220 kDa) in myofibrillar protein enriched fractions was similar in PH and controls (Fig. 5A). We also found that sarcomeric actin (Fig. 5B) or troponin T (Fig. 5C) were unchanged in whole muscle homogenates (Western blots).

## Discussion

The main findings of our study were that PH caused diaphragm atrophy and impaired both isometric and isotonic contractile properties in mice. Moreover, food consumption of PH mice was unchanged despite a progressive decrease in body weight over 6–8 weeks. These alterations in contractile function occurred in the absence of increases in protein oxidation or decreases in myofibrillar protein abundance.

Patients with PH experience loss of body weight, although the exact prevalence is unknown [4]. Patients with PH who experience loss of body weight have increased mortality rate after lung transplantation [31]. Loss of muscle mass is an important component of loss of body weight in chronic diseases [32]–[34]. Accordingly, soleus muscle mass of PH mice was 15% lower than control. The decreases in body and muscle weight were not caused by differences in food intake. In contrast, rats show ∼78% decrease in food intake 20 days after a single MCT injection compared to control [14]. This is an important observation when considering the experimental design of studies using MCT in rodents. To our knowledge, there are no published data showing that PH patients become anorexic, and it appears that PH per se does not cause anorexia in patients [18]. Our study shows that loss of body weight in PH mice is accompanied by decreases in muscle mass, and these changes are not caused by anorexia. Hence, our findings suggest that mice can be used to investigate the mechanisms of diaphragm contractile dysfunction in PH without confounding effects of anorexia induced by MCT.

Patients with PH have a 25–35% lower maximal inspiratory pressure than healthy peers [6], [35], which suggests diaphragm weakness. This weakness can arise from fiber atrophy and contractile dysfunction. Previous studies found diaphragm fiber atrophy in rats and patients [3]. Moreover, there was diminished specific force (force normalized for cross sectional area) in diaphragm bundles of rats with PH induced by MCT [3]. The decrease in specific force was evident in permeabilized single fibers of rats and patients with PH [3], [7], showing that PH causes dysfunction of the contractile apparatus.

In agreement with previous studies [3], [7], we found diaphragm fiber atrophy and decreased submaximal and maximal specific isometric tension in PH mice. The effects of PH on the diaphragm force-frequency relationship were slightly different in mice and rats. In mice, force was depressed predominantly at high stimulus frequencies (>50 Hz). In rats, the depression of force occurred at all frequencies [3]. These discrepancies might reflect the hypersensitivity of rats to MCT. The important point, however, is that mice injected with MCT to cause PH show diminished isometric specific tetanic force as seen in patients with PH. Specific force (reported here in kN/m^2^) represents force normalized for bundle cross sectional area, thus a decrease in specific force suggests contractile dysfunction independent of changes in muscle or fiber size. Thus, the ability of the diaphragm to generate force (or pressure) in PH is dimished due to combined effects of decreased fiber size (atrophy) and contractile dysfunction.

The force-frequency characteristics provide useful information regarding the force generating capacity of diaphragm, but existing data are limited to isometric contractions. The diaphragm rarely performs isometric contractions *in vivo* [25]. Rather, it shortens against submaximal loads. Therefore, shortening velocity and power output are more physiologically relevant properties of diaphragm function. Patients with PH show a 20% decrease in maximal sniff nasal pressure [6] – a volitional dynamic maneuver that involves diaphragm shortening. Similarly, maximal voluntary ventilation is 30% lower in patients with primary PH than in healthy controls [2]. The decrease in sniff nasal pressure and maximal voluntary ventilation in PH patients suggest, among other factors, impairment in isotonic contractile properties of the diaphragm. Indeed, we found 40% slower V_max_ and 60% lower peak power in the diaphragm of mice with PH. There was also a downward shift in the force-velocity relationship when submaximal loads were normalized for maximal tetanic force, e.g., Fig. 3A. Because we used afterloaded isotonic contractions, the downward shift reflects slower cross-bridge kinetics due to factors intrinsic to cross-bridge cycling or the level of calcium activation [24]. Importantly, afterloaded isotonic tests simulate the contractile profile *in vivo*, and our data supports the notion that PH impairs the ability of the diaphragm to sustain ventilatory tasks. Impairment of isotonic function could, then, contribute to dyspnea in PH. However, the mechanisms underlying contractile dysfunction are unclear.

Oxidants decrease calcium release and calcium sensitivity of the contractile apparatus [36], [37], and impair sarcomeric protein function [38]. However, PH did not change protein oxidation status measured by abundance of carbonyls in whole-diaphragm homogenates. Our results corroborate the data from a recent study showing unchanged protein carbonyls in PH rats [9]. Administration of MCT in rats increases protein carbonyls in limb muscles [16]. Thus, protein oxidation may be specific of limb muscles or become apparent after the development of right ventricular failure. Additionally, we cannot discard the possibility that PH increases other oxidant-mediated modifications (e.g., thiol oxidation). Based on the current and recent data [16], we consider that PH-induced diaphragm isometric and isotonic contractile dysfunctions are not caused by increased protein oxidation.

A mechanism that has been proposed to explain diaphragm weakness in chronic diseases is selective degradation of myofibrillar proteins [39]–[41]. A recent study has shown that MHC abundance was unchanged in rats with PH induced by MCT [16], which is consistent with our data in mice. Loss of thin-filament proteins could also contribute to contractile dysfunction. PH elevates systemic levels of pro-inflammatory cytokines [17]. The pro-inflammatory cytokine interleukin 1 promotes degradation of sarcomeric actin [42], which would cause contractile dysfunction. Tumor necrosis factor-α stimulates selective degradation of troponin T that results in diminished isometric force [43]. We measured the abundance of actin and troponin T and found that both were unchanged in MCT mice. Thus, our findings substantiate the concept that selective degradation of myofibrillar proteins does not appear cause contractile dysfunction in PH [7]. Rather, post-translational modifications are the likely cause of isometric and isotonic contractile impairments in PH. Identification of these post-translational modifications was beyond the scope of our investigation, but PH may affect the phosphorylation or thiol oxidation status of myofibrillar proteins [44]–[46].

### Conclusion

Our conclusion is that MCT-induced PH causes diaphragm atrophy and impairs diaphragm isometric and isotonic contractile properties in mice. These perturbations in isotonic diaphragm contractile properties likely play a role in impaired ventilatory responses of PH patients [47]. Contractile dysfunction in PH mice occurs in the absence of selective degradation of myofibrillar proteins or accumulation of protein carbonyls, which suggests post-translational modifications of myofibrillar proteins such as phosphorylation and thiol oxidation as alternative mechanisms for contractile impairments. Importantly, MCT causes loss of body weight but not anorexia.

Hence, our findings suggest that mice can be used to investigate the mechanisms of diaphragm muscle weakness and atrophy in PH without confounding effects of anorexia induced by MCT.

## References

[1] Humbert M, Khaltaev N, Bousquet J, Souza R (2007) Pulmonary hypertension: from an orphan disease to a public health problem. Chest 132: 365–367. 17699126 10.1378/chest.07-0903

[2] Sun XG, Hansen JE, Oudiz RJ, Wasserman K (2003) Pulmonary function in primary pulmonary hypertension. J Am Coll Cardiol 41: 1028–1035. 12651053 10.1016/s0735-1097(02)02964-9

[3] de Man FS, van Hees HW, Handoko ML, Niessen HW, Schalij I, et al (2011) Diaphragm muscle fiber weakness in pulmonary hypertension. Am J Respir Crit Care Med 183: 1411–1418. 21131469 10.1164/rccm.201003-0354OC

[4] le Roux CW, Ghatei MA, Gibbs JS, Bloom SR (2005) The putative satiety hormone PYY is raised in cardiac cachexia associated with primary pulmonary hypertension. Heart 91: 241–242. 15657252 10.1136/hrt.2003.026880 PMC1768707

[5] Anker SD, Ponikowski P, Varney S, Chua TP, Clark AL, et al (1997) Wasting as independent risk factor for mortality in chronic heart failure. Lancet 349: 1050–1053. 9107242 10.1016/S0140-6736(96)07015-8

[6] Kabitz HJ, Schwoerer A, Bremer HC, Sonntag F, Walterspacher S, et al (2008) Impairment of respiratory muscle function in pulmonary hypertension. Clin Sci (Lond) 114: 165–171. 17764445 10.1042/CS20070238

[7] Manders E, de Man FS, Handoko ML, Westerhof N, van Hees HW, et al (2012) Diaphragm weakness in pulmonary arterial hypertension: role of sarcomeric dysfunction. Am J Physiol Lung Cell Mol Physiol 303: L1070–1078. 22962018 10.1152/ajplung.00135.2012

[8] Kanj NA, Nasser MG, Medawar WA, Al Tayeh AU, Khoury MY, et al (1999) Reversal of impaired calcium homeostasis in the rat diaphragm subjected to Monocrotaline-induced pulmonary hypertension. Toxicol Lett 105: 177–182. 10355538 10.1016/s0378-4274(98)00398-1

[9] Degens H, Bosutti A, Gilliver SF, Slevin M, van Heijst A, et al (2010) Changes in contractile properties of skinned single rat soleus and diaphragm fibres after chronic hypoxia. Pflugers Arch 460: 863–873. 20697736 10.1007/s00424-010-0866-5

[10] Cho YJ, Han JY, Lee SG, Jeon BT, Choi WS, et al (2009) Temporal changes of angiopoietins and Tie2 expression in rat lungs after monocrotaline-induced pulmonary hypertension. Comp Med 59: 350–356. 19712575 PMC2779210

[11] Dumitrascu R, Koebrich S, Dony E, Weissmann N, Savai R, et al (2008) Characterization of a murine model of monocrotaline pyrrole-induced acute lung injury. BMC Pulm Med 8: 25. 19087359 10.1186/1471-2466-8-25 PMC2635347

[12] Molteni A, Ward WF, Ts'ao CH, Solliday NH (1989) Monocrotaline pneumotoxicity in mice. Virchows Arch B Cell Pathol Incl Mol Pathol 57: 149–155. 2570481 10.1007/BF02899076

[13] Nishii Y, Gabazza EC, Fujimoto H, Nakahara H, Takagi T, et al (2006) Protective role of protein C inhibitor in monocrotaline-induced pulmonary hypertension. J Thromb Haemost 4: 2331–2339. 17059470 10.1111/j.1538-7836.2006.02174.x

[14] Steffen BT, Lees SJ, Booth FW (2008) Anti-TNF treatment reduces rat skeletal muscle wasting in monocrotaline-induced cardiac cachexia. J Appl Physiol 105: 1950–1958. 18801959 10.1152/japplphysiol.90884.2008

[15] Yamazato Y, Ferreira AJ, Hong KH, Sriramula S, Francis J, et al (2009) Prevention of pulmonary hypertension by Angiotensin-converting enzyme 2 gene transfer. Hypertension 54: 365–371. 19564552 10.1161/HYPERTENSIONAHA.108.125468 PMC2732127

[16] Dalla Libera L, Ravara B, Gobbo V, Danieli Betto D, Germinario E, et al (2005) Skeletal muscle myofibrillar protein oxidation in heart failure and the protective effect of Carvedilol. J Mol Cell Cardiol 38: 803–807. 15850574 10.1016/j.yjmcc.2005.02.023

[17] Vescovo G, Zennaro R, Sandri M, Carraro U, Leprotti C, et al (1998) Apoptosis of skeletal muscle myofibers and interstitial cells in experimental heart failure. J Mol Cell Cardiol 30: 2449–2459. 9925379 10.1006/jmcc.1998.0807

[18] Rubenfire M, Bayram M, Hector-Word Z (2007) Pulmonary hypertension in the critical care setting: classification, pathophysiology, diagnosis, and management. Crit Care Clin 23: 801–834, vi–vii. 10.1016/j.ccc.2007.07.006 17964364

[19] Ismail HM (2007) Reversible pulmonary hypertension and isolated right-sided heart failure associated with hyperthyroidism. J Gen Intern Med 22: 148–150. 17351857 10.1007/s11606-006-0032-0 PMC1824731

[20] Qin L, D'Alessandro-Gabazza CN, Aoki S, Gil-Bernabe P, Yano Y, et al (2010) Pulmonary hypertension is ameliorated in mice deficient in thrombin-activatable fibrinolysis inhibitor. J Thromb Haemost 8: 808–816. 20088932 10.1111/j.1538-7836.2010.03751.x

[21] Nih/Od/Oer/Olaw (2010) Guide for the Care and Use of Laboratory Animals: Eighth Edition: National Academies Press. 246 p.

[22] Senf SM, Dodd SL, McClung JM, Judge AR (2008) Hsp70 overexpression inhibits NF-kappaB and Foxo3a transcriptional activities and prevents skeletal muscle atrophy. FASEB J 22: 3836–3845. 18644837 10.1096/fj.08-110163 PMC6137947

[23] Reed SA, Sandesara PB, Senf SM, Judge AR (2012) Inhibition of FoxO transcriptional activity prevents muscle fiber atrophy during cachexia and induces hypertrophy. FASEB J 26: 987–1000. 22102632 10.1096/fj.11-189977 PMC3289501

[24] Bullimore SR, Saunders TJ, Herzog W, MacIntosh BR (2010) Calculation of muscle maximal shortening velocity by extrapolation of the force-velocity relationship: afterloaded versus isotonic release contractions. Can J Physiol Pharmacol 88: 937–948. 20962893 10.1139/y10-068

[25] van Hees HW, van der Heijden HF, Hafmans T, Ennen L, Heunks LM, et al (2008) Impaired isotonic contractility and structural abnormalities in the diaphragm of congestive heart failure rats. Int J Cardiol 128: 326–335. 17689734 10.1016/j.ijcard.2007.06.080

[26] Machiels HA, van der Heijden HF, Heunks LM, Dekhuijzen PN (2001) The effect of hypoxia on shortening contractions in rat diaphragm muscle. Acta Physiol Scand 173: 313–321. 11736693 10.1046/j.1365-201X.2001.00895.x

[27] Close RI (1972) Dynamic properties of mammalian skeletal muscles. Physiol Rev 52: 129–197. 4256989 10.1152/physrev.1972.52.1.129

[28] Hill AV (1938) The Heat of Shortening and the Dynamic Constants of Muscle. Proceedings of the Royal Society of London Series B – Biological Sciences 126: 136–195. 10.1098/rspb.1949.0019 18152150

[29] Sakamoto K, Hirshman MF, Aschenbach WG, Goodyear LJ (2002) Contraction regulation of Akt in rat skeletal muscle. J Biol Chem 277: 11910–11917. 11809761 10.1074/jbc.M112410200

[30] Layland J, Cave AC, Warren C, Grieve DJ, Sparks E, et al (2005) Protection against endotoxemia-induced contractile dysfunction in mice with cardiac-specific expression of slow skeletal troponin I. FASEB J. 19: 1137–1139. 10.1096/fj.04-2519fje 15855227

[31] Habedank D, Ewert R, Hetzer R, Anker SD (2009) Reversibility of cachexia after bilateral lung transplantation. Int J Cardiol 133: 46–50. 18234375 10.1016/j.ijcard.2007.11.077

[32] Hasselgren PO, Fischer JE (2001) Muscle cachexia: current concepts of intracellular mechanisms and molecular regulation. Ann Surg 233: 9–17. 11141219 10.1097/00000658-200101000-00003 PMC1421177

[33] Tisdale MJ (2002) Cachexia in cancer patients. Nat Rev Cancer 2: 862–871. 12415256 10.1038/nrc927

[34] Fearon KC, Glass DJ, Guttridge DC (2012) Cancer cachexia: mediators, signaling, and metabolic pathways. Cell Metab 16: 153–166. 22795476 10.1016/j.cmet.2012.06.011

[35] Meyer FJ, Lossnitzer D, Kristen AV, Schoene AM, Kubler W, et al (2005) Respiratory muscle dysfunction in idiopathic pulmonary arterial hypertension. Eur Respir J 25: 125–130. 15640333 10.1183/09031936.04.00095804

[36] Andrade FH, Reid MB, Allen DG, Westerblad H (1998) Effect of hydrogen peroxide and dithiothreitol on contractile function of single skeletal muscle fibres from the mouse. J Physiol 509 (Pt 2): 565–575. 10.1111/j.1469-7793.1998.565bn.x PMC2230964 9575304

[37] Moopanar TR, Allen DG (2005) Reactive oxygen species reduce myofibrillar Ca2+ sensitivity in fatiguing mouse skeletal muscle at 37 degrees C. J Physiol. 564: 189–199. 10.1113/jphysiol.2005.083519 PMC1456045 15718257

[38] Callahan LA, She ZW, Nosek TM (2001) Superoxide, hydroxyl radical, and hydrogen peroxide effects on single-diaphragm fiber contractile apparatus. J Appl Physiol 90: 45–54. 11133892 10.1152/jappl.2001.90.1.45

[39] Argadine HM, Hellyer NJ, Mantilla CB, Zhan WZ, Sieck GC (2009) The effect of denervation on protein synthesis and degradation in adult rat diaphragm muscle. J Appl Physiol 107: 438–444. 19520837 10.1152/japplphysiol.91247.2008 PMC2724326

[40] van Hees HW, Li YP, Ottenheijm CA, Jin B, Pigmans CJ, et al (2008) Proteasome inhibition improves diaphragm function in congestive heart failure rats. Am J Physiol Lung Cell Mol Physiol 294: L1260–1268. 18424622 10.1152/ajplung.00035.2008 PMC3101573

[41] Ottenheijm CA, Heunks LM, Dekhuijzen RP (2008) Diaphragm adaptations in patients with COPD. Respir Res 9: 12. 18218129 10.1186/1465-9921-9-12 PMC2248576

[42] Li W, Moylan JS, Chambers MA, Smith J, Reid MB (2009) Interleukin-1 stimulates catabolism in C2C12 myotubes. Am J Physiol Cell Physiol 297: C706–714. 19625606 10.1152/ajpcell.00626.2008 PMC2740393

[43] Adams V, Mangner N, Gasch A, Krohne C, Gielen S, et al (2008) Induction of MuRF1 is essential for TNF-alpha-induced loss of muscle function in mice. J Mol Biol 384: 48–59. 18804115 10.1016/j.jmb.2008.08.087

[44] Solaro RJ, Kobayashi T (2011) Protein phosphorylation and signal transduction in cardiac thin filaments. J Biol Chem 286: 9935–9940. 21257760 10.1074/jbc.R110.197731 PMC3060547

[45] Canton M, Neverova I, Menabo R, Van Eyk J, Di Lisa F (2004) Evidence of myofibrillar protein oxidation induced by postischemic reperfusion in isolated rat hearts. Am J Physiol Heart Circ Physiol 286: H870–877. 14766672 10.1152/ajpheart.00714.2003

[46] Canton M, Skyschally A, Menabo R, Boengler K, Gres P, et al (2006) Oxidative modification of tropomyosin and myocardial dysfunction following coronary microembolization. Eur Heart J 27: 875–881. 16434410 10.1093/eurheartj/ehi751

[47] Schwaiblmair M, Faul C, von Scheidt W, Berghaus TM (2012) Ventilatory efficiency testing as prognostic value in patients with pulmonary hypertension. BMC Pulm Med 12: 23. 22676304 10.1186/1471-2466-12-23 PMC3420250

